# Targeting PI3K and AMPKα Signaling Alone or in Combination to Enhance Radiosensitivity of Triple Negative Breast Cancer

**DOI:** 10.3390/cells9051253

**Published:** 2020-05-19

**Authors:** Jeremy Johnson, Zeta Chow, Dana Napier, Eun Lee, Heidi L. Weiss, B. Mark Evers, Piotr Rychahou

**Affiliations:** 1 Department of Toxicology and Cancer Biology, University of Kentucky, Lexington, KY 40536, USA; jeremy-johnson@uky.edu; 2 Markey Cancer Center, University of Kentucky, Lexington, KY 40536, USA; zeta.chow@uky.edu (Z.C.); dana.napier@uky.edu (D.N.); heidi.weiss@uky.edu (H.L.W.); mark.evers@uky.edu (B.M.E.); 3 Department of Surgery, University of Kentucky, Lexington, KY 40536, USA; 4 Department of Pathology and Laboratory Medicine, University of Kentucky, Lexington, KY 40536, USA; eylee@uky.edu

**Keywords:** triple negative breast cancer, radiation, radiosensitivity, AMPK, PI3K

## Abstract

Triple negative breast cancer (TNBC) is the most aggressive breast cancer subtype and is characterized by poor survival. Radiotherapy plays an important role in treating TNBC. The purpose of this study was to determine whether inhibiting the AMP-activated protein kinase (AMPK) and phosphatidylinositol 3-kinase (PI3K) pathways alone or in combination potentiates radiotherapy in TNBC. AMPKα1 and AMPKα2 knockdown diminished cyclin D1 expression and induced G1 cell cycle arrest but did not induce apoptosis alone or in combination with radiotherapy. Next, we analyzed the role of PI3K p85α, p85β, p110α, p110β, Akt1, and Akt2 proteins on TNBC cell cycle progression and apoptosis induction. Akt1 and p110α knockdown diminished cyclin D1 expression and induced apoptosis. Silencing Akt1 promoted synergistic apoptosis induction during radiotherapy and further reduced survival after radiation. Treatment with the Akt inhibitor, MK-2206 48 h after radiotherapy decreased Akt1 levels and potentiated radiation-induced apoptosis. Together, our results demonstrate that AMPKα, p110α, and Akt1 promote TNBC proliferation and that Akt1 is a key regulator of radiosensitivity in TNBC. Importantly, combining radiotherapy with the pharmacological inhibition of Akt1 expression is a potentially promising approach for the treatment of TNBC.

## 1. Introduction

Breast cancer is the most common and second deadliest cancer among women worldwide [1]. The most aggressive subtype is triple negative breast cancer (TNBC), which makes up about 15%–20% of cases [1,2]. TNBC is characterized by a lack of expression of the estrogen receptor, progesterone receptor, and HER2 [1,3]. It affects a younger patient population, metastasizes at a higher rate than other subtypes, and has a poor prognosis [1,2]. Tumor analysis has revealed four separate subtypes of TNBC [4]. Due to this heterogeneity, the development of targeted therapies for TNBC has largely been unsuccessful [5]. Standard approaches involve chemotherapy, which can be combined with surgery and/or radiation, but improvements still need to be made [1,2]. Radiosensitization, which involves combining radiotherapy with an agent that can potentiate its effects, is a potential advancement that warrants further study in TNBC.

Radiation improves survival in TNBC patients [6,7] by inducing single- and double-stranded DNA breaks through direct DNA damage or via the generation of free radicals [8,9,10]. Radiated cells respond to this by activating stress, survival, and metabolic pathways, and repair enzymes attempt to fix damaged DNA [8,9,10]. If the DNA is not successfully repaired, then the apoptotic cascade will be induced and lead to cell death [8,9,10]. Blocking cellular pathways that control metabolism or survival may prevent cells from adequately repairing damaged DNA, thereby enhancing apoptosis induction and leading to radiosensitization. Two proteins that play important roles in metabolism and survival are AMP-activated protein kinase (AMPK) and phosphatidylinositol 3-kinase (PI3K), respectively. Furthermore, a double-negative feedback loop exists between AMPK and Akt, a major downstream effector of PI3K, in breast cancer [11].

AMPK is a heterotrimer that is made up of a catalytic subunit (α) and two regulatory subunits (β and γ) [12,13,14,15,16,17,18,19]. The catalytic subunit consists of α1 and α2 isoforms [12,13,14,15,16,17,18,19]. AMPK is a major metabolic regulator that is activated during low energy states and helps cells return to energy homeostasis by increasing ATP production and reducing ATP consumption [12,13,14,15,16,17,18,19]. Radiotherapy induces cellular stress and has led to AMPK activation in cancer cells [20,21]. Moreover, AMPK has provided a survival benefit in esophageal and nasopharyngeal carcinoma cells exposed to radiation by mediating autophagy induction [21,22]. Furthermore, levels of phosphorylated and total AMPK were upregulated in radiation-resistant colon cancer cells and tissue with AMPK knockdown sensitizing radiation-resistant cells to radiotherapy [23]. PI3K is a heterodimer that promotes cellular growth and survival by activating Akt, which subsequently activates the mammalian target of rapamycin (mTOR) [24,25,26,27,28]. PI3K is composed of a catalytic subunit (p110α or p110β) and a regulatory subunit (p85α or p85β) [24,25,26]. Akt is composed of three isoforms, namely Akt1, Akt2, and Akt3 [27,28]. The PI3K/Akt/mTOR pathway is the most altered pathway in breast cancer, and almost 30% of TNBC cases have PI3K alterations [29,30]. Studies have indicated that 20%–25% of these changes were due to mutations in *PIK3CA*, which is the gene for p110α [29,30]. Since this signaling cascade promotes survival, it has also been targeted in efforts to induce radiosensitivity. Chemical inhibition of PI3K or mTOR signaling—alone or in combination—sensitized estrogen receptor-positive breast cancer and TNBC to radiotherapy [31,32,33,34,35]. In addition, ectopic expression of Akt1 promoted radioresistance in estrogen receptor-positive breast cancer [36]. However, targeting individual AMPK isoforms, PI3K subunits, or Akt isoforms—alone or in combination—to enhance the effects of radiation in TNBC has not previously been done.

In the present study, we investigated the role of AMPK and the PI3K/Akt pathway in TNBC radiation sensitivity and examined whether combination therapy can improve treatment response to radiation therapy. We determined that both Akt1 and p110α promote proliferation and prevent apoptosis in TNBC cells. Next, we established that knockdown of Akt1 enhances radiation-induced apoptosis in TNBC cells and decreases survival of TNBC cells after radiation. Knockdown of AMPKα1 or AMPKα2 did not enhance radiation-induced apoptosis alone or in combination with Akt1 knockdown. Finally, we showed that treatment with the Akt inhibitor MK-2206 reduces Akt1 expression and promotes synergistic apoptosis induction with radiotherapy in a schedule-dependent manner. Targeting Akt1 expression at the initiation of radiation-induced apoptosis could improve TNBC patient outcomes, reduce toxicity associated with PI3K/mTOR inhibition, and mitigate side effects from radiotherapy.

## 2. Materials and Methods

### 2.1. Materials

Roswell Park Memorial Institute (RPMI) 1640 medium was from Thermo Fisher (Waltham, MA, The Netherlands). Dulbecco’s Modified Eagle Medium (DMEM) was from Corning (Corning, NY, USA). DMEM/F12 was from Sigma-Aldrich (St. Louis, MO, USA). Fetal bovine serum (FBS), 100× penicillin–streptomycin solution (PS), 10 mg/mL insulin solution, 100× non-essential amino acid solution, and Dulbecco’s phosphate buffered saline (PBS) were from Sigma-Aldrich. Opti-MEM and Lipofectamine RNAiMAX were from Thermo Fisher. The protein assay dye was from Bio-Rad (Hercules, CA, USA). Amersham ECL Prime Western blotting detection reagent was from GE Healthcare Life Sciences (Chicago, IL, USA). Immobilon Western chemiluminescent HRP substrate was from Millipore (Burlington, MA, USA). Pooled siRNAs for AMPKα1 and AMPKα2 were from Santa Cruz Biotechnology (Dallas, TX, USA). Pooled siRNAs for Akt1, Akt2, p110α, p110β, p85α, and p85β were from Thermo Fisher. The Cytoscan™ SRB cell cytotoxicity assay was from G-Biosciences (St. Louis, MO, USA). The propidium iodide flow cytometry kit for cell cycle analysis was from Abcam (Boston, MA, USA). MK-2206 2HCl, A-674563, AZD5363, and perifosine were from Cayman Chemical (Ann Arbor, MI, USA). Primary antibodies used in this study include Abcam: (1) AMPKα1, ab32047 (1:1000 for WB, 1:100 for IHC for PDXs, and 1:175 for IHC for whole tissue sections), (2) AMPKα2, ab3760 (1:1000 for WB, 1:200 for IHC for PDXs, and 1:300 for IHC for whole tissue sections), (3) cyclin D1, ab134175 (1:2500 for WB); Cell Signaling Technology (Danvers, MA, USA): (1) pAkt, #4060 (1:2000 for WB), (2) Akt1, #75692 (1:1000 for WB), (3) Akt2, #3063 (1:1000 for WB), (4) p110α, #4249 (1:1000 for WB), (5) p110β, #3011 (1:1000 for WB), (6) cleaved PARP, #5625 (1:1000 for WB), (7) caspase-3, #9664 (1:1000 for WB), (8) p53, #2527 (1:1000 for WB), (9) pDNA-PKcs, #68716 (1:1000 for WB), (10) DNA-PKcs, #12311 (1:1000 for WB), (11) pAMPKα, #50081 (1:1000 for WB), and (12) AMPKα, #5831 (1:1000 for WB); Santa Cruz Biotechnology: (1) p85α, sc-71891 (1:1000 for WB), (2) p85β, sc-515646 (1:1000 for WB); and Sigma-Aldrich: β-actin (1:10,000 for WB). Secondary antibodies were from Santa Cruz Biotechnology.

### 2.2. Cell Culture

MDA-MB-175, MDA-MB-231, MDA-MB-453, MDA-MB-468, HCC-1143, HCC-1806, BT-20, BT-549, and HS-578T cells were from the American Type Culture Collection. All cells were cultured in a humidified incubator at 37 °C and 5% CO_2_. MDA-MB-231, MDA-MB-453, HCC-1143, and HCC-1806 cells were cultured in RPMI-1640 + 10% FBS + 1% PS. BT-549 cells were cultured in RPMI-1640 + 10 µg/mL insulin + 10% FBS + 1% PS. HS-578T cells were cultured in DMEM + 10 µg/mL insulin + 10% FBS + 1% PS. BT-20 cells were cultured in DMEM + 1× amino acids + 10% FBS + 1% PS. MDA-MB-175 cells were cultured in DMEM + 10% FBS + 1% PS. MDA-MB-468 cells were cultured in DMEM/F12 + 10% FBS + 1% PS.

### 2.3. siRNA Transfection

TNBC cells were transfected with siRNA to NTC, AMPKα1, AMPKα2, p110α, p110β, p85α, p85β, Akt1, or Akt2. The final siRNA concentration used in each experiment is indicated in the relevant descriptions. The siRNAs were mixed with lipofectamine RNAiMAX in Opti-MEM for 20 min. The ratio of Opti-MEM to complete medium was 1:4. Medium was changed every 24 h until cell lysis or subsequent seeding for downstream applications.

### 2.4. Radiation Treatment

(I) In time-course and dose-response experiments, MDA-MB-231 cells were irradiated at the indicated doses with an X-RAD 225XL (Precision X-Ray, North Branford, CT, USA) at the X-ray Service Center of the Department of Toxicology and Cancer Biology. The X-ray beam setting was 225 kVp with 0.3 mm Cu filtration. The beam output was calibrated following AAPM TG-61 protocol with measurement done in-air. An A1SL ion chamber (Standard Imaging, Madison, WI, USA) was used, which was calibrated at the University of Wisconsin Accredited Dosimetry Calibration Laboratory (UW ADCL) in terms of air kerma (Gy/C). Accurate absorbed doses to water were calculated by considering the impact of backscattering as previously described [37]. Cells were lysed for immunoblotting at the indicated times. (II) To establish radiation-induced apoptosis induction, two methods were used. The first was a single radiation dose (0, 6, or 10 Gy) followed by incubation at 37 °C and 5% CO_2_ for 48 h or 72 h before lysis. The second was a fractionated approach in which cells were irradiated at the same dose (0, 2, 4, or 6 Gy) daily for 4 d with lysis occurring 24 h after the last dose. (III) MDA-MB-231 cells were transfected with siRNA to NTC, AMPKα1, Akt1, or AMPKα1/Akt1. Transfection concentrations were (1) individual siRNA: 50 nM, (2) combination siRNA: 50 nM each (100 nM total), and (3) siNTC: 100 nM. Cells were irradiated at the indicated doses either on the same day as transfection or 24 h after transfection. Cells were incubated at 37 °C and 5% CO_2_ for 72 h after transfection before lysis for immunoblotting.

### 2.5. Analysis of Glycolytic Rate

MDA-MB-231 cells were transfected with 50 nM siRNA to NTC, AMPKα1, or AMPKα2. Medium was changed after 24 h, and cells were seeded into Seahorse XF96 cell culture microplates at a density of 20,000 cells per well after 48 h. At 72 h post transfection, the Redox Metabolism Shared Resource Facility at the University of Kentucky measured the glycolytic rate with a Seahorse XFe96 analyzer (Agilent, Santa Clara, CA, USA). The glycolytic rate assay was conducted according to the manufacturer’s protocol [38]. Cells were initially treated with 0.5 μM rotenone and 0.5 μM antimycin A before a final injection of 50 mM 2-deoxy-d-glucose. Analysis was performed with the Agilent Seahorse Wave Desktop software. Measurements of the glycolytic rate were based on the glycolytic proton efflux rate (glycoPER). The buffer factor was set to 2.4 mmol/L/pH, and data were normalized to μG protein in each well.

### 2.6. Immunoblotting

TNBC cells were seeded and transfected as described above. Final concentrations were 50 nM or 100 nM, as indicated in the appropriate figures. After 72 h, medium was removed, and cells were washed with ice cold 1× PBS. Cells were then scraped and lysed in 1× radioimmunoprecipitation assay (RIPA) buffer containing 1 mM phenylmethylsulfonyl fluoride (PMSF). Cells were incubated on ice for 20 min with 10-sec vortexes every 5 min before centrifugation at 14,000× *g* for 20 min at 4 °C. Protein concentrations in the lysates were then determined. Equal amounts of protein were reduced and denatured by heating at 80 °C for 10 min before being resolved on 4%–12% Bis–Tris gels. The proteins were then transferred to polyvinylidene fluoride (PVDF) membranes, blocked with 10% milk for at least 1 h, and incubated in primary antibody solutions overnight at 4°C. On the next day, the membranes were washed twice with 1× Tris-buffered saline with Tween 20 (TBST) for 5 min and 10 min before incubation with secondary antibody solutions (1:10,000 dilutions) for 1 h at room temperature. The membranes were then washed twice with TBST for 15 min and 20 min before Amersham ECL or Immobilon were added to the membranes for protein detection. Stripping buffer was used on membranes where required.

To determine apoptosis induction after radiation, the above procedure was modified. First, to include floating cells that had undergone apoptosis, the medium at 48 h post transfection was saved and frozen at 80 °C until cell lysis. At the time of lysis, cells were scraped before medium removal, combined with the previously frozen medium, and centrifuged at 14,000× *g* for 5 min at 4 °C. The medium was then suctioned off, and the remaining pellet was washed with 1× PBS and centrifuged at 14,000× *g* for 5 min at 4 °C. After removing the PBS, the cells were lysed with 1× RIPA buffer containing 1 mM PMSF as described above.

### 2.7. Cell Counting Assay

MDA-MB-231 cells were transfected with 50 nM siRNA to NTC, AMPKα1, or AMPKα2 as described above. Medium was changed after 24 h. After 48 h, cells were washed with 1× PBS, trypsinized, and counted with a Beckman Coulter Vi-Cell XR. Then equal numbers of each transfected cell (0.1 × 10^6^ cells per well) were seeded in 6-well plates and incubated under normal cell culture conditions. Medium was changed after 72 h, and cell counting was performed after 96 h with the same instrument.

### 2.8. Sulforhodamine B (SRB) Assay

MDA-MB-231 cells were transfected with siRNA to NTC, AMPKα1, AMPKα2, Akt1, or p110α (including combinations). Transfection concentrations were (1) individual siRNA: 50 nM, (2) combination siRNA: 50 nM each (100 nM total), and (3) siNTC: 100 nM. Medium was changed after 24 h, and equal numbers of each transfected cell (3000 cells per well) were seeded in 96-well plates after 48 h. Cells were allowed to incubate under normal cell culture conditions for 48 h. Cells were then fixed, stained, and quantified following the Cytoscan™ SRB cell cytotoxicity assay protocol.

### 2.9. Colony Formation Assay

MDA-MB-231 cells were transfected with siRNA to NTC, AMPKα1, Akt1, or AMPKα1/Akt1. Transfection concentrations were (1) individual siRNA: 50 nM, (2) combination siRNA: 50 nM each (100 nM total), and (3) siNTC:100 nM. After 48 h, cells were seeded at equal density in 96-well plates (100 cells/well). Cells were then exposed to radiation (0 or 4 Gy) on the following day. After 7 days, cells were fixed, stained, and quantified following the SRB assay protocol described above.

### 2.10. Flow Cytometry

MDA-MB-231 cells were transfected with 50 nM siRNA to NTC, AMPKα1, or AMPKα2 as described above. Medium was changed after 24 h, and cells were seeded into separate 10-cm plates after 48 h. On the following day, cells were collected, fixed in 66% ethanol, and stored at 4 °C for at least 2 h. Before analysis, cells were rehydrated in PBS and stained with a solution containing propidium iodide and RNase for 30 min at 37 °C in the dark. Analysis of DNA content was performed by measuring the propidium iodide fluorescence intensity with a flow cytometer in the Flow Cytometry and Immune Monitoring Core at the University of Kentucky.

### 2.11. Immunohistochemistry

TNBC whole tissue samples were selected by the Markey Cancer Center Biospecimen Core. Four micrometer slides were deparaffinized and hydrated stepwise. Antigen retrieval was carried out in a Biocare Medical decloaking chamber at 95 °C for 20 min, followed by quenching of endogenous peroxidase activity and incubation with primary antibody overnight at 4 °C. The slides were subsequently incubated with Vector Laboratories ImmPRESS^®^ anti-rabbit HRP polymer for 30 min at room temperature and staining was visualized with DAB (Dako). Antibody specific conditions were as follows: (1) AMPKα1: Dako high pH antigen retrieval buffer; 10-min DAB incubation; 1:175 dilution and (2) AMPKα2: Dako low pH antigen retrieval buffer; 5 min DAB incubation; 1:300 dilution. A pathologist who was blinded to the stage of disease scored the samples for staining intensity and distribution percentage on scales from 0 to 3. For staining intensity: 0 = negative; 1 = weak; 2 = moderate; and 3 = strong. For distribution percentage: 0 = 0%; 1 = 1%–10%; 2 = 11%–50%; and 3 = 51%–100%.

TNBC patient-derived xenografts (PDXs) were from Dr. Kathleen O’Connor’s laboratory and were immunostained as described above except the AMPKα1 dilution was 1:100 and the AMPKα2 dilution was 1:200.

### 2.12. Statistical Analysis

Descriptive statistics including means and standard deviations (SD) are presented in each experimental group and displayed in bar graphs. Comparisons of WB, proliferation, oxidative stress markers, and SRB absorbance were performed using the one sample t-test, one way and two-way analysis of variance (ANOVA) with Holm’s adjustment for multiple testing between groups. For the combination studies, a two-way ANOVA with factors for AMPKα1/AMPKα2 knockdown and Akt1 or p110α treatments along with interaction between factors was used. Likewise, two-way ANOVA with an interaction term was utilized to account for the differential effect of radiation in the comparison of AMPKα1/AMPKα2 knockdown and Akt1 single and combination groups. *p* < 0.05 was considered to indicate a statistically significant difference. Statistical analyses were performed using SAS software version 9.4 (SAS Inc., Cary, NC, USA).

## 3. Results

### 3.1. Analysis of AMPKα1 and AMPKα2 Expression in TNBC Patient Samples, Cell Lines and PDXs

**Figure 1:**
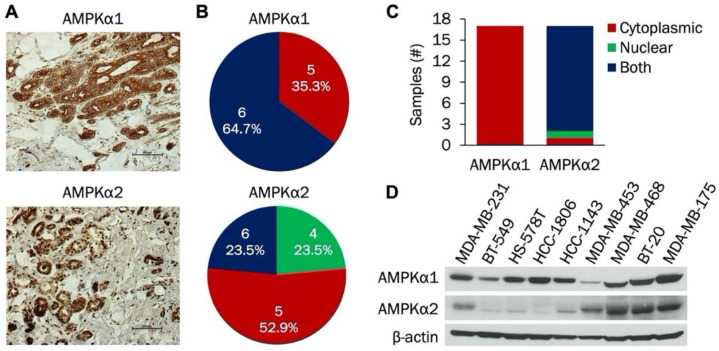
Analysis of AMPKα1 and AMPKα2 expression in triple negative breast cancer (TNBC) whole tissue sections and established cell lines. (**A**) Representative immunohistochemical staining of AMPKα1 and AMPKα2 in TNBC whole tissue sections. (**B**) Scoring distribution of AMPKα1 and AMPKα2 in TNBC whole tissue sections (*n* = 17). (**C**) Subcellular localization (nuclear, cytoplasmic, or both) of AMPKα1 and AMPKα2 in TNBC whole tissue sections (*n* = 17). (**D**) Western blot of AMPKα1 and AMPKα2 expression in a panel of established TNBC cell lines (*n* = 9).

AMPK is an important regulator of cellular metabolism that has recently gained attention as a potential target in cancer therapy. The catalytic subunit is composed of α1 and α2 isoforms; expression of the individual isoforms has not been studied in TNBC. Therefore, we initially examined expression of AMPKα1 and AMPKα2 isoforms in TNBC patient samples, cell lines, and PDXs. Representative IHC staining of both AMPKα1 and AMPKα2 in patient samples is shown in Figure 1A. In these images, AMPKα1 was expressed in the cytoplasm while AMPKα2 was expressed in both the nucleus and cytoplasm. Figure 1B indicates the scoring distribution of AMPKα1 and AMPKα2 among the patient samples. Each sample received a score of 4, 5, or 6 for both AMPKα1 and AMPKα2, indicating strong and/or widespread expression in each tumor. Next, cellular localization of AMPKα1 and AMPKα2 was compared in these patient samples. As shown in Figure 1C, AMPKα1 was expressed only in the cytoplasm, while AMPKα2 was found in both the cytoplasm and the nucleus. Immunoblotting was then used to study the expression of both isoforms in a panel of TNBC cell lines (Figure 1D). AMPKα1 was expressed in all cell lines with the lowest level in MDA-MB-453 cells, but AMPKα2 expression was more variable. MDA-MB-231, MDA-MB-453, MDA-MB-468, BT-20, and MDA-MB-175 cells had high AMPKα2 expression, while BT-549, HS-578T, HCC-1806, and HCC-1143 cells had low AMPKα2 expression.

We extended our analysis to include AMPKα1 and AMPKα2 expression in TNBC PDX samples. Representative IHC staining of AMPKα1 and AMPKα2 in TNBC PDX samples is shown in Supplementary Materials Figure S1A. In these images, AMPKα1 was expressed in the cytoplasm while AMPKα2 was found in both the nucleus and cytoplasm. The scoring distribution of AMPKα1 and AMPKα2 in the PDX samples is indicated in Supplementary Materials Figure S1B. All samples were scored as 4, 5, or 6, signifying strong and/or widespread expression. Analysis of isoform localization was also done. As shown in Supplementary Materials Figure S1C, AMPKα1 was found only in the cytoplasm, while AMPKα2 was expressed in both the nucleus and the cytoplasm. Taken together, our results indicate that AMPKα1 and AMPKα2 are widely expressed in TNBC with AMPKα2 predominantly localized to the nucleus.

### 3.2. AMPKα1 and AMPKα2 Promote TNBC Proliferation and Cell Cycle Progression

**Figure 2:**
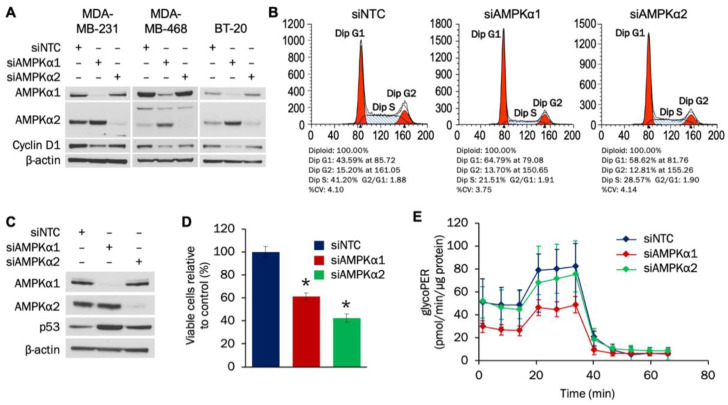
AMPKα1 and AMPKα2 impact proliferation and cell cycle progression in TNBC cells. (**A**) MDA-MB-231, MDA-MB-468, and BT-20 cells were transfected with 50 nM of siRNA targeting AMPKα1 or AMPKα2. Expression of AMPKα1, AMPKα2, and cyclin D1 was analyzed by Western blot 72 h after transfection. (**B**) MDA-MB-231 cells were transfected with 50 nM of siRNA targeting AMPKα1 or AMPKα2. Cells were seeded into 10-cm plates 48 h after transfection and collected for cell cycle analysis 24 h later. (**C**) MDA-MB-231 cells were transfected with 50 nM of siRNA targeting AMPKα1 or AMPKα2. Expression of AMPKα1, AMPKα2, and p53 was analyzed by Western blot 72 h after transfection. (**D**) MDA-MB-231 cells were transfected with 50 nM of siRNA targeting AMPKα1 or AMPKα2. Cells were plated at a density of 100,000 cells/well 48 h after transfection and counted 96 h after plating. (**E**) MDA-MB-231 cells were transfected with 50 nM of siRNA targeting AMPKα1 or AMPKα2. Cells were plated at a density of 20,000 cells/well 48 h after transfection, and the glycolytic rate was analyzed 24 h later (*n* = 18). For 2A–D, the results are representative of 3 independent experiments. For 2E, the result is representative of 2 independent experiments. * Indicates *p*-value < 0,0001. NTC (non-targeting control) was used as a negative control.

The individual roles of AMPKα1 and AMPKα2 isoforms in TNBC are not well understood. Therefore, we examined how each isoform impacts proliferation and metabolism in TNBC cells. Using siRNA transfection, levels of both AMPKα1 and AMPKα2 were successfully reduced after 72 h in MDA-MB-231, MDA-MB-468, and BT-20 cells (Figure 2A). Knockdown of either isoform led to decreased cyclin D1 levels in MDA-MB-231 and MDA-MB-468 cells, while only AMPKα1 knockdown resulted in cyclin D1 suppression in BT-20 cells. Densitometry analysis indicated statistically significant reductions in cyclin D1 with AMPKα1 knockdown in all three cell lines and with AMPKα2 knockdown in MDA-MB-231 and MDA-MB-468 cells (Supplementary Materials Figure S2A).

We next determined whether AMPKα regulates cell cycle and proliferation in MDA-MB-231 cells. Flow cytometry analysis established that knockdown of either AMPKα1 or AMPKα2 led to G1 cell cycle arrest (Figure 2B). The tumor suppressor p53 can prevent cells from progressing past G1, and knockdown of either AMPKα1 or AMPKα2 led to increased expression of p53 (Figure 2C). Densitometry analysis indicated that the increases in p53 were statistically significant (Supplementary Materials Figure S2B). To evaluate whether the induced G1 cell cycle arrest impacted MDA-MB-231 growth, a proliferation assay was then done. As shown in Figure 2D, knockdown of either AMPKα1 or AMPKα2 resulted in statistically significant decreases in MDA-MB-231 proliferation.

Glycolytic activity increases during the G1 phase, and AMPK has been shown to affect the cellular glycolytic rate [39,40,41]. Therefore, the effect that AMPKα1 or AMPKα2 knockdown had on glycolysis in MDA-MB-231 cells was determined. Figure 2E indicates that knockdown of AMPKα1—but not AMPKα2—decreased the glycolytic rate in MDA-MB-231 cells. The decreases in glycolytic flux with AMPKα1 knockdown were statistically significant for both basal and compensatory glycolysis (Supplementary Materials Figure S3A–B). Taken together, our results indicate that both AMPKα1 and AMPKα2 promote cell cycle progression and proliferation of MDA-MB-231 cells by potentially downregulating p53 expression, while AMPKα1 also facilitates glycolytic flux.

### 3.3. Knockdown of Akt1 or p110α Induces Apoptosis and Reduces Proliferation in MDA-MB-231 Cells

**Figure 3:**
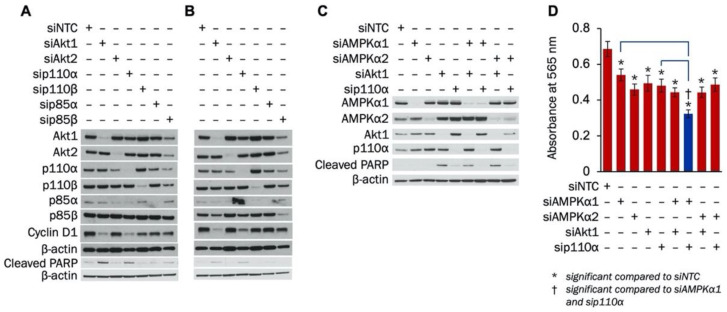
Knockdown of Akt1 or p110α increases apoptosis and decreases proliferation in MDA-MB-231 cells. MDA-MB-231 cells were transfected with 100 nM of siRNA to Akt1, Akt2, p110α, p110β, p85α, or p85β. Expression of Akt1, Akt2, p110α, p110β, p85α, p85β, cyclin D1, and cleaved PARP was determined with Western blot (**A**) 72 h and (**B**) 96 h after transfection. (**C**) MDA-MB-231 cells were transfected with siRNA to AMPKα1, AMPKα2, Akt1, or p110α alone or in combinations. Transfection concentrations were (1) individual siRNA: 50 nM, (2) combination siRNA: 50 nM each (100 nM total), and (3) siNTC: 100 nM. Expression of AMPKα1, AMPKα2, Akt1, p110α, and cleaved PARP was determined with Western blot 72 h after transfection. (**D**) MDA-MB-231 cells were transfected with siRNA to AMPKα1, AMPKα2, Akt1, or p110α alone or in combinations. Transfection concentrations were (1) individual siRNA: 50 nM, (2) combination siRNA: 50 nM each (100 nM total), and (3) siNTC: 100 nM. Cells were plated at a density of 3000 cells/well 48 h after transfection. SRB growth assay was performed 48 h after plating (*n* = 12). *indicates *p*-value < 0.0001; NTC, Non-targeting control was used as a negative control.

The impact of individual PI3K subunits or Akt isoforms on TNBC proliferation or apoptosis has not been studied. The effect of combined inhibition of PI3K and AMPKα on TNBC growth and survival is also unknown. Therefore, we examined how knockdown of PI3K signaling components—alone or in combination with AMPKα isoforms—affected apoptosis induction and proliferation in MDA-MB-231 cells. Initially, siRNA was used to establish knockdown of p110α, p110β, p85α, p85β, Akt1, and Akt2. Figure 3A,B show siRNA knockdown of all components at 72 h and 96 h, respectively. Knockdown of all proteins except p85β was accomplished at 72 h (Figure 3A), while knockdown of p85β was confirmed at 96 h (Figure 3B). At both 72 h and 96 h, suppression of Akt1 and p110α led to the largest decreases in cyclin D1 and the most substantial increases in cleaved PARP.

Next, the effect of combined knockdown of PI3K and AMPKα on apoptosis induction and proliferation in MDA-MB-231 cells was determined. As shown in Figure 3C, PARP cleavage was induced with Akt1 knockdown alone or in combination with knockdown of AMPKα1 or AMPKα2 in MDA-MB-231 cells. Slight increases in PARP cleavage were also found with knockdown of p110α by itself or when combined with knockdown of AMPKα2. In addition, an SRB growth assay was performed to establish whether combined knockdown of AMPKα isoforms with Akt1 or p110α suppressed MDA-MB-231 proliferation more than knockdown of each protein alone. Single knockdown of AMPKα1, AMPKα2, Akt1, or p110α significantly reduced MDA-MB-231 growth (Figure 3D). Additionally, MDA-MB-231 proliferation was further decreased when knockdown of AMPKα1 was combined with knockdown of either Akt1 or p110α. However, the growth of MDA-MB-231 cells was not further suppressed when AMPKα2 knockdown was combined with knockdown of either Akt1 or p110α. Taken together, our results indicate that silencing either Akt1 or p110α induces apoptosis and suppresses proliferation of MDA-MB-231 cells, and combination knockdown with AMPKα1 further reduces growth.

### 3.4. Radiotherapy Increases Activated and Total AMPKα in MDA-MB-231 Cells

**Figure 4:**
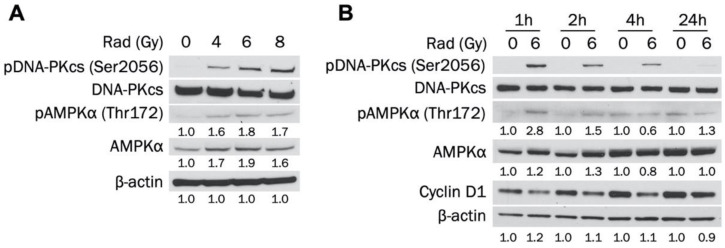
Radiation increases levels of activated and total AMPKα in MDA-MB-231 cells. (**A**) MDA-MB-231 cells were exposed to 0, 2, 4, or 6 Gy radiation dose and collected 1 h after irradiation. Total and phosphorylated AMPKα and DNA-PKcs expression levels were determined by Western blot. (**B**) MDA-MB-231 cells were exposed to 0 or 6 Gy radiation dose and collected 1 h, 2 h, 4 h, or 24 h after irradiation. Total and phosphorylated AMPKα and DNA-PKcs expression levels were determined by Western blot.

AMPK has been activated in cancer cells by radiation and has increased survival through the autophagy pathway [20,21,22]. However, the ability of radiotherapy to activate AMPK in TNBC in unknown. Therefore, we established whether radiation leads to AMPKα activation in MDA-MB-231 cells. Figure 4A indicates that AMPKα was activated 1 h after exposure to increasing doses of radiation (4, 6, and 8 Gy). AMPKα was activated to a similar degree at each dose, indicating that the effect was not dose-dependent. In addition, levels of total AMPKα were increased at every dosage. DNA-dependent protein kinase (DNA-PK) is a DNA repair enzyme that was also activated at each radiation dose. Figure 4B shows that 6 Gy radiation activated AMPK in MDA-MB-231 cells 1 h and 2 h post exposure, with the largest increase at 1 h. However, pAMPK levels returned to baseline at 4 h and were not elevated at 24 h. Total AMPKα was also slightly increased 1 h and 2 h after radiation treatment. DNA-PK phosphorylation was detected after 1 h and decreased in a stepwise fashion at 2 h, 4 h, and 24 h post exposure. Taken together, our results indicate that radiation increases activated and total levels of AMPKα in a non-dose-dependent manner in MDA-MB-231 cells.

### 3.5. Akt1 Knockdown Potentiates Radiation-Induced Apoptosis and Further Suppresses Survival after Radiation in MDA-MB-231 Cells

**Figure 5:**
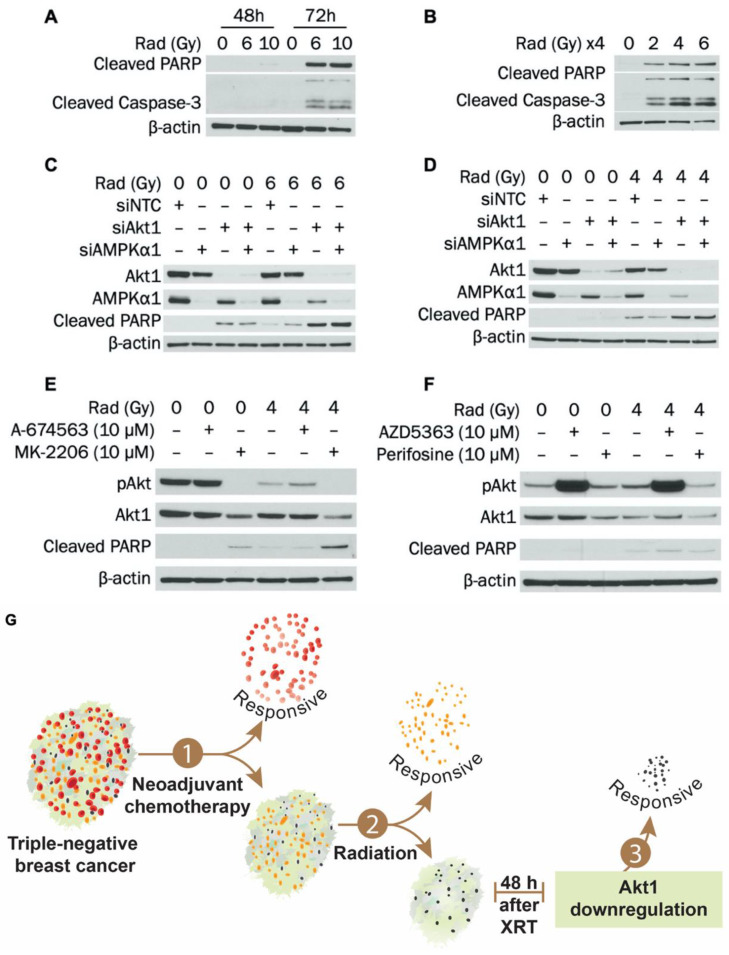
Knockdown of Akt1 potentiates radiation-induced apoptosis in MDA-MB-231 cells. (**A**) MDA-MB-231 cells were exposed to 0, 6, or 10 Gy radiation dose and collected 48 h or 72 h after irradiation. Expression of cleaved PARP and cleaved caspase-3 was determined by Western blot. (**B**) MDA-MB-231 cells were exposed to 0, 2, 4, or 6 Gy radiation dose daily for 4 d and collected 24 h after the last radiation treatment. Expression of cleaved PARP and cleaved caspase-3 was determined by Western blot. (**C**) MDA-MB-231 cells were transfected with siRNA to AMPKα1, Akt1, or AMPKα1/Akt1. Transfection concentrations were (1) individual siRNA: 50 nM, (2) combination siRNA: 50 nM each (100 nM total), and (3) NTC: 100 nM. Cells were exposed to 0 or 6 Gy radiation dose 24 h after transfection and collected 48 h after radiation. Expression of AMPKα1, Akt1, and cleaved PARP was determined by Western blot. (**D**) MDA-MB-231 cells were exposed to 0 or 4 Gy radiation dose and then transfected with siRNA to AMPKα1, Akt1, or AMPKα1/Akt1 on the same day. Transfection concentrations were (1) individual siRNA: 50 nM, (2) combination siRNA: 50 nM each (100 nM total), and (3) NTC: 100 nM. Cells were collected 72 h after transfection. Expression of AMPKα1, Akt1, and cleaved PARP was determined by Western blot. (**E**) MDA-MB-231 cells were exposed to 0 or 4 Gy radiation dose and then incubated for 48 h. Cells were then treated with 0 or 10 μM of A-674563 or MK-2206 for 24h. Expression of pAkt, Akt1, and cleaved PARP was determined by Western blot. (**F**) MDA-MB-231 cells were exposed to 0 or 4 Gy radiation dose and then incubated for 48 h. Cells were then treated with 0 or 10 μM of AZD5363 or perifosine for 24 h. Expression of pAkt, Akt1, and cleaved PARP was determined by Western blot. (**G**) Summary diagram showing Akt1 inhibition and radiation-induced apoptosis in TNBC cells. For A–D, NTC (non-targeting control) was used as a negative control.

Radiotherapy causes DNA damage, stress, and apoptosis. However, activation of AMPK and Akt may help cells adapt to—and eventually overcome—these stressful conditions. Therefore, we evaluated how knockdown of AMPKα1 and Akt1—alone or in combination—impacts radiation-induced apoptosis in MDA-MB-231 cells. Attempts were initially made to determine when apoptosis is induced in MDA-MB-231 cells following radiotherapy. Two approaches were used: (1) a single dose of radiation followed by lysis after 48 h or 72 h or (2) a fractionated approach in which cells were radiated at the same dose for 4 consecutive days and harvested 24 h after the last treatment (96 h after the first dose). As shown in Figure 5A, a single dose of 6 or 10 Gy induced cleavage of PARP and caspase-3 after 72 h in MDA-MB-231 cells. In addition, a single dose of 10 Gy slightly induced PARP cleavage after 48 h. In Figure 5B, fractionated dosing (2, 4, or 6 Gy daily for 4 d) also led to strong PARP and caspase-3 cleavage after 96 h total in MDA-MB-231 cells.

To determine whether combined knockdown of AMPKα1 and Akt1 potentiates radiation-induced apoptosis, a single radiation dosing scheme similar to that in Figure 5A was used instead of the fractionated approach. Figure 5C indicates that 6 Gy radiation induced PARP cleavage after 48 h and that this was substantially enhanced when combined with 72 h Akt1 knockdown. However, knockdown of AMPKα1 alone or in combination with Akt1 did not enhance this effect. These findings were verified, as seen in Supplementary Materials Figure S4A. As shown in Figure 5D, similar results were observed when radiating with 4 Gy and incubating for 72 h. PARP cleavage was induced with 4 Gy radiation alone after 72 h but was enhanced when radiation was combined with 72 h Akt1 knockdown. As with the 48 h timepoint, AMPKα1 knockdown alone or in combination with Akt1 did not potentiate the increases in PARP cleavage. The effect of Akt1 silencing on MDA-MB-231 survival after radiation was also examined. Either Akt1 knockdown or treatment with 4 Gy radiation significantly reduced colony formation of MDA-MB-231 cells, and survival was further suppressed in radiated cells with Akt1 knockdown (Supplementary Materials Figure S4B). Knockdown of AMPKα1 slightly attenuated the effect of Akt1 knockdown at both 0 Gy and 4 Gy. Taken together, our results indicate that silencing Akt1 sensitizes MDA-MB-231 cells to radiation-induced apoptosis and decreases survival after radiotherapy.

### 3.6. MK-2206 Treatment Enhances Radiation-Induced Apoptosis in MDA-MB-231 Cells

To increase the clinical relevance of our findings, we examined the effect of Akt inhibitors MK-2206, A-674563, AZD5363 and perifosine on apoptosis induction in MDA-MB-231 cells after radiation. Cells were treated with Akt inhibitors 48 h after radiation exposure (Figure 5E,F). Treatment with MK-2206 alone for 24 h reduced Akt1 expression and induced PARP cleavage in MDA-MB-231 cells. In cells that were exposed to 4 Gy radiation, treatment with MK-2206 synergistically increased PARP cleavage compared to either modality alone. Administration of perifosine also reduced Akt1 expression but did not synergistically increase radiation-induced apoptosis. Treatment with other Akt inhibitors—A-674563 or AZD5363—did not reduce Akt1 levels or induce apoptosis. Taken together, our results indicate that reducing Akt1 expression with MK-2206 at the initiation of apoptosis can potentiate the effect of radiotherapy. Targeting Akt1 expression as a radiosensitizer in TNBC could enhance patient outcomes and mitigate toxicity associated with both PI3K pathway inhibition and radiotherapy.

## 4. Discussion

Triple-negative breast cancer (TNBC) (estrogen receptor-negative, progesterone receptor-negative, and HER2-negative) is an aggressive subgroup of breast cancer [1,2]. Treating patients with TNBC remains clinically challenging; radiation therapy is able to improve locoregional control in breast cancer patients both after breast conserving surgery or mastectomy, with positive impact on long-term survival [6,7]. Radiotherapy’s importance as a treatment modality in TNBC prompted our interest in attempting to further increase survival advantage of radiotherapy in TNBC patients. We targeted two important cellular pathways, AMPK and PI3K/Akt, with the hypothesis that their combined inhibition would potentiate radiation-induced apoptosis.

The expression and function of individual AMPKα isoforms in TNBC has not previously been studied. We demonstrated that both AMPKα1 and AMPKα2 were expressed in TNBC patient samples, cell lines, and PDXs. AMPKα1 was localized to the cytoplasm, while AMPKα2 was expressed in both the cytoplasm and the nucleus. AMPKα2 expression has been shown to be enriched in the nucleus compared to AMPKα1 in rat insulinoma cells [42]. AMPKα controls cellular proliferation and glycolytic flux in TNBC [40,41], but distinctions have not been made between the two isoforms. We found that both AMPKα1 and AMPKα2 promote proliferation and cell cycle progression of MDA-MB-231 cells. We also determined that knockdown of either AMPKα1 or AMPKα2 led to upregulation of p53, a tumor suppressor that can induce cell cycle arrest. Although silencing AMPKα2 in BT-20 cells did not reduce cyclin D1 expression, we suspect that this may be due to insufficient knockdown. Our densitometry calculations indicated a trend toward reduced cyclin D1 expression in BT-20 cells with AMPKα2 knockdown, and we expect that a more robust knockdown would yield results consistent with our other cell lines. Taken together, our results suggest that AMPKα1 and AMPKα2 support proliferation of MDA-MB-231 cells by potentially reducing p53 levels. In other cancer cells, increased AMPK activity or expression has led to amplified p53 levels [43,44]. AMPK has a complex cellular role that can vary based on the method by which AMPK is manipulated. For instance, TNBC growth can be suppressed by AMPK activation or with stable AMPKα knockdown [40,45]. Therefore, we attribute the difference in our findings to using a knockdown model instead of drug-induced AMPK activation or overexpression. To our knowledge, no studies have established a link between knockdown of endogenous AMPKα expression and p53 levels in cancer cells. Further work is needed to establish the mechanism by which AMPKα1 or AMPKα2 regulates p53 expression in TNBC cells. Knockdown of AMPKα1 did not enhance radiation-induced apoptosis, suggesting that inhibiting the AMPKα pathway does not provide further benefit. However, there may be value in examining the combination of AMPKα activation and radiation in TNBC. Other studies have demonstrated an advantage of AMPKα activation during radiotherapy [46,47,48,49]. In particular, metformin—an AMPKα activator—has been shown to increase cell death after radiation exposure in lung, esophageal, and estrogen receptor-positive breast cancer cells [46,47,48]. This is partly due to AMPK’s ability to inhibit mTOR signaling, so a combination approach with Akt1 knockdown could be examined [47,48,49]. Future work could evaluate the ability of increased AMPKα activity—whether by genetic upregulation or chemical activation—to sensitize TNBC cells to radiation.

The PI3K/Akt/mTOR pathway is altered more frequently than any other pathway in breast cancer [30]. However, the effect of individual subunits or isoforms on proliferation or apoptosis induction in TNBC is unknown. We found that knockdown of either Akt1 or p110α reduced cyclin D1 expression and increased cleaved PARP expression in TNBC cells. The other components—Akt2, p110β, p85α, and p85β—had minimal effects on cyclin D1 expression or PARP cleavage. We selected Akt1 as our main target for combination therapy with radiation, because its knockdown alone induced a higher rate of apoptosis compared to p110α knockdown. Other studies have also found that Akt1 is the most important Akt isoform at inhibiting apoptosis induction in breast cancer and mouse myeloid cells [50,51]. Suppressing Akt1 expression during radiotherapy could be a valuable approach in treating TNBC that is refractory to neoadjuvant chemotherapy (NAC). TNBC is initially treated with NAC to shrink the tumor as much as possible before surgery. Radiotherapy may be initiated after surgery in an attempt to induce death of resistant cells. Our data indicate that suppressing Akt1 levels—possibly with MK-2206—may sensitize resistant TNBC tumors to radiotherapy. To our knowledge, this is the first study to establish a link between Akt1 expression and radiation-induced apoptosis in TNBC. This strategy could reduce future recurrence and ultimately lead to better outcomes for TNBC patients.

Apoptosis is one of the primary methods by which radiotherapy induces cell death [10]. We demonstrate that the apoptotic cascade is not immediately activated after radiation and it takes up to 48 h after radiation therapy to detect apoptosis in TNBC cells. Akt inhibition or knockdown right after radiation therapy do not synergistically increase the effect of radiation therapy. However, we show the potential for the selective sensitization of tumor cells to radiation therapy with timed knockdown of Akt1 expression after radiation therapy. MK-2206 promoted synergistic apoptosis induction when administered after radiotherapy, and we expect drugs that strongly reduce Akt1 expression (similar to genetic knockdown) will promote radiation-induced apoptosis to an even greater degree. The ability to enhance the effect of radiation therapy by reducing Akt1 levels at the time of initial radiation-induced apoptosis induction is a particularly significant finding and should be of relevance to the design of clinical combination protocols. By timing Akt inhibitor administration with radiation therapy, we can reduce the frequency of Akt inhibitor administration and the amount of drug given to the patient with TNBC. To our knowledge, this is the first study to identify that an Akt inhibitor can be administered in a schedule-dependent manner to induce radiosensitivity in TNBC cells. Selective targeting of Akt1 protein expression should also decrease the severity of side effects associated with PI3K/Akt pathway inhibition. In addition, reducing Akt1 expression to enhance radiotherapy may lessen the number of treatments required to induce apoptosis or arrest tumor growth, potentially making radiation more tolerable for TNBC patients.

In summary (Figure 5G), we established that silencing Akt1 sensitized TNBC cells to radiation treatment. Knockdown of Akt1 potentiated apoptosis induction after radiotherapy and further suppressed TNBC cell survival after treatment. Administration of MK-2206 at the initiation of radiation-induced apoptosis reduced Akt1 expression and enhanced radiation therapy effect in TNBC cells. Importantly, combining radiotherapy with pharmacological inhibition of Akt1 expression at the optimal timing is a potentially promising approach for the treatment of TNBC that could improve TNBC response to radiotherapy and limit toxicity associated with PI3K pathway inhibition.

## Supplementary Materials

The following are available online at https://www.mdpi.com/2073-4409/9/5/1253/s1, Figures S1–S4 contain graphs, a Western blot, and IHC staining that is referenced in the manuscript, while Data Tables S1–S5 contain data that was used for calculations in the manuscript.
