# Supplementary material for: Targeting PI3K and AMPKα Signaling Alone or in Combination to Enhance Radiosensitivity of Triple Negative Breast Cancer
Source: Cells. 2020 May 19;9(5):1253. doi: 10.3390/cells9051253 (PMC7291172; doi:10.3390/cells9051253)
Supplement: Supplementary file 1 [file cells-09-01253-s001.pdf]

Targeting PI3K and AMPKα Signaling Alone or in Combination to Enhance Radiosensitivity of Triple Negative Breast Cancer

Jeremy Johnson [1], Zeta Chow [2,3], Dana Napier [2], Eun Lee [4], Heidi L. Weiss [2],

B. Mark Evers [2,3] and Piotr Rychahou [2,3,]†

[1] Department of Toxicology and Cancer Biology, [2] Markey Cancer Center, [3] Department of Surgery, [4] Department of Pathology and Laboratory Medicine, Lexington, KY 40536, USA

†**Corresponding Author:** Piotr Rychahou, M.D., University of Kentucky, Markey Cancer Center, 760 Press Ave, HKRB 322, Lexington, KY 40536, (859) 323-9285,

piotr.rychahou@uky.edu

SUPPLEMENTARY MATERIALS

Figures S1-S4 contain graphs and IHC staining that is referenced in the manuscript.

Data Tables S1-S5 contain data that was used for calculations in the manuscript.

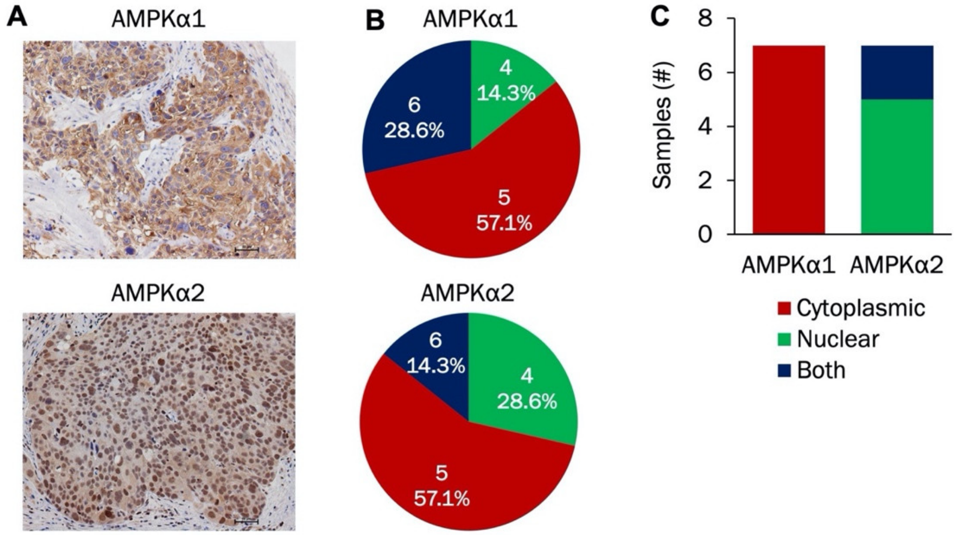

Figure S1. Analysis of AMPKα1 and AMPKα2 expression in TNBC PDX samples. (A) Representative IHC staining of AMPKα1 and AMPKα2 in TNBC PDX samples. **(B)** Scoring distribution of AMPKα1 and AMPKα2 in TNBC PDX samples ($n = 7$). **(C)** Subcellular localization (nuclear, cytoplasmic, or both) of AMPKα1 and AMPKα2 in TNBC PDX samples ($n = 7$).

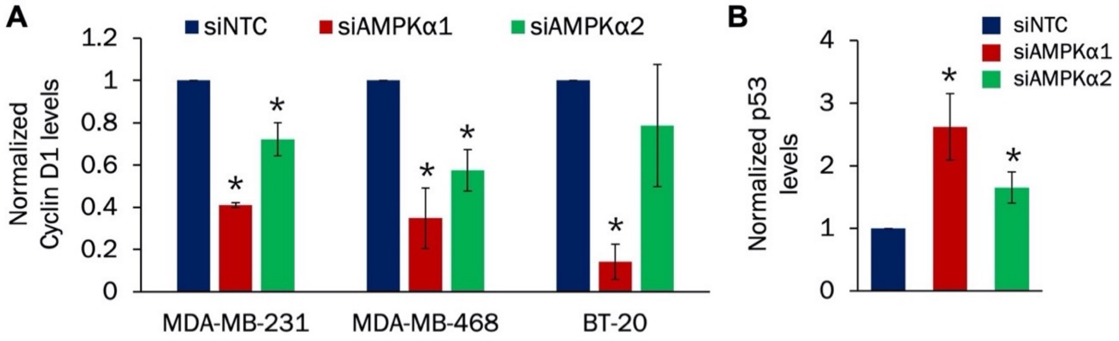

Figure S2. Densitometry measurements of cyclin D1 and p53 after AMPKα1 or AMPKα2 knockdown in TNBC cells. (A) MDA-MB-231, MDA-MB-468, and BT-20 cells were transfected with 50 nM of siRNA targeting AMPKα1 or AMPKα2. Expression of cyclin D1 was analyzed by western blot densitometry 72h after transfection ($n = 3$). **(B)** MDA-MB-231 cells were transfected with 50 nM of siRNA targeting AMPKα1 or AMPKα2. Expression of p53 was analyzed by western blot densitometry 72h after transfection ($n = 3$). *indicates p-value < 0.05; NTC, Non-targeting control was used as a negative control.

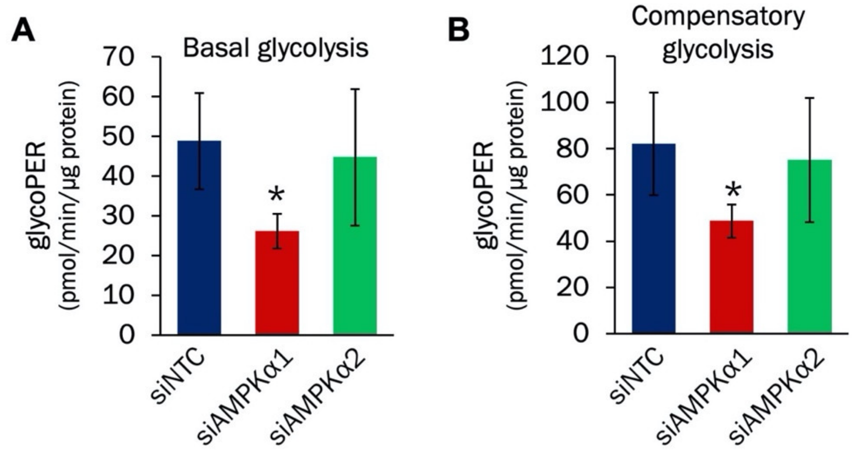

Figure S3. Effect of AMPKα1 or AMPKα2 knockdown on basal and compensatory glycolysis in MDA-MB-231 cells. MDA-MB-231 cells were transfected with 50 nM of siRNA targeting AMPKα1 or AMPKα2. Non-targeting control was used as a negative control. Cells were seeded at a density of 20,000 cells/well 48h after transfection, and the rates of (A) basal and (B) compensatory glycolysis were determined 24h later (n = 18). The results are representative of 2 independent experiments. *indicates p-value < 0.0001.

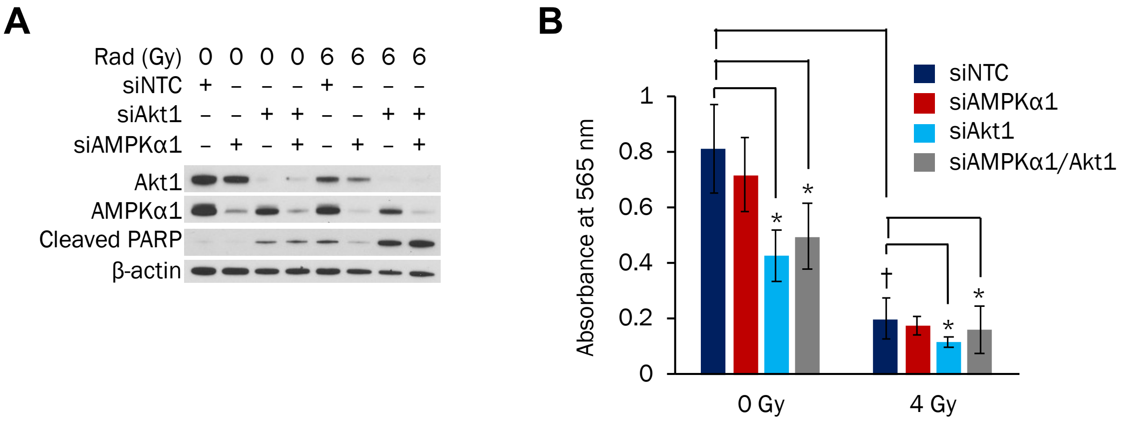

Figure S4. Silencing Akt1 enhances radiation-induced apoptosis in MDA-MB-231 cells. (A) MDA-MB-231 cells were transfected with siRNA to AMPKα1, Akt1, or AMPKα1/Akt1. Transfection concentrations were: (1) individual siRNA: 50 nM, (2) combination siRNA: 50 nM each (100 nM total), and (3) NTC: 100 nM. Cells were exposed to 0 or 6 Gy radiation dose 24h after transfection and collected 48h after radiation. Expression of AMPKα1, Akt1, and cleaved PARP was determined by western blot. **(B)** MDA-MB-231 cells were transfected with siRNA to AMPKα1, Akt1, or AMPKα1/Akt1. Transfection concentrations were: (1) individual siRNA: 50 nM, (2) combination siRNA: 50 nM each (100 nM total), and (3) NTC: 100 nM. Cells were

seeded at a density of 100 cells/well into 96-well plates 48h after transfection. Cells were exposed to 0 or 4 Gy radiation dose 24h later. Colonies were fixed and quantified with the SRB assay protocol 1 week after irradiation ($n = 18$). NTC, Non-targeting control was used as a negative control. *indicates $p < 0.01$ vs. group control; † indicates $p < 0.0001$ vs. no radiation.

Data Table S1. Western blot data used for densitometry calculations in paper.

Data Table S1. Western blot data used for densitometry calculations in paper.

MDA-MB-231 Cells			
	Normalized Cyclin D1		
	siNTC	siAMPKα1	siAMPKα2
Rep 1	1	0.42207499	0.80997237
Rep 2	1	0.407942915	0.69800853
Rep 3	1	0.399301194	0.6577587
	Normalized p53		
	siNTC	siAMPKα1	siAMPKα2
Rep 1	1	3.05383243	1.86973381
Rep 2	1	2.780327811	1.71017351
Rep 3	1	2.031290802	1.37851921

MDA-MB-468 Cells			
	Normalized Cyclin D1		
	siNTC	siAMPKα1	siAMPKα2
Rep 1	1	0.24249245	0.48132773
Rep 2	1	0.29001615	0.56460761
Rep 3	1	0.51194717	0.67725438

BT-20 Cells			
	Normalized Cyclin D1		
	siNTC	siAMPKα1	siAMPKα2
Rep 1	1	0.05161267	0.78855112
Rep 2	1	0.21674908	1.07579107
Rep 3	1	0.15322177	0.49636264

Data Table S2. Basal and compensatory glycolysis data used for calculations in paper.

Data Table S2. Basal and compensatory glycolysis data used for calculations in paper.

Basal Glycolysis (pmol/min) Normalized to Protein			Compensatory Glycolysis (pmol/min) Normalized to Protein		
siNTC	siAMPKα1	siAMPKα2	siNTC	siAMPKα1	siAMPKα2
39.757	26.270	49.452	75.048	54.395	80.078
35.704	29.472	43.687	61.667	53.728	69.214
35.005	36.071	75.691	59.746	65.136	127.037
52.317	21.988	37.145	85.600	44.852	62.676
35.670	25.915	34.467	62.588	45.060	67.187
36.571	27.885	46.620	68.281	52.579	70.114
56.372	22.188	51.353	95.911	37.083	82.375
41.575	24.592	31.961	41.101	44.570	50.939
38.938	26.279	40.141	67.702	48.317	67.357
55.327	23.724	91.778	96.115	43.631	151.227
57.050	32.007	23.021	93.248	55.723	38.091
58.685	33.598	44.753	94.672	58.125	74.312
48.206	22.931	48.244	86.205	40.909	78.137
56.313	19.778	25.377	81.919	41.546	47.238
42.964	27.575	31.796	73.923	51.058	61.436
42.894	25.092	59.557	77.372	51.531	91.670
67.971	21.618	33.194	123.878	45.730	66.763
77.320	23.762	37.468	131.254	41.653	66.342

Data Table S3. Proliferation assay data used for calculations in paper.

Data Table S3. Proliferation assay data used for calculations in paper.

	Viable cells /ml (x10^6)	Viable cells relative to siNTC		Viable cells /ml (x10^6)	Viable cells relative to siNTC		Viable cells /ml (x10^6)	Viable cells relative to siNTC
siNTC	1.04	103.9170776	siAMPKα1	0.59	59.22782154	siAMPKα2	0.44	44.09728361
siNTC	1.05	105.1211074	siAMPKα1	0.64	64.39508242	siAMPKα2	0.40	40.37485197
siNTC	0.98	98.14781374	siAMPKα1	0.57	57.4318239	siAMPKα2	0.37	37.24439708
siNTC	0.94	93.94377435	siAMPKα1	0.63	63.08068955	siAMPKα2	0.44	43.73607705
siNTC	0.99	98.87022687	siAMPKα1	0.63	63.08068955	siAMPKα2	0.46	46.61569737
	Average							
siNTC	1.00							

Data Table S4. SRB assay data used for calculations in paper.

Data Table S4. SRB assay data used for calculations in paper.

	siNTC	siAMPKα1	siAMPKα2	siAkt1	sip110α	siAMPKα1/Akt1	siAMPKα1/p110α	siAMPKα2/Akt1	siAMPKα2/p110α
	0.605	0.516	0.442	0.435	0.472	0.446	0.312	0.398	0.443
	0.661	0.538	0.434	0.481	0.43	0.455	0.337	0.428	0.475
	0.64	0.55	0.503	0.532	0.491	0.471	0.361	0.437	0.541
	0.649	0.493	0.481	0.484	0.453	0.494	0.348	0.449	0.5
	0.656	0.502	0.476	0.529	0.446	0.461	0.337	0.52	0.541
	0.717	0.52	0.434	0.478	0.449	0.418	0.328	0.452	0.525
	0.694	0.584	0.481	0.418	0.488	0.43	0.306	0.43	0.451
	0.71	0.512	0.396	0.453	0.498	0.431	0.339	0.456	0.456
	0.716	0.55	0.49	0.481	0.564	0.439	0.3	0.441	0.482
	0.724	0.578	0.453	0.531	0.513	0.442	0.292	0.421	0.458
Absorbance	0.745	0.595	0.48	0.537	0.464	0.436	0.316	0.453	0.44
at 565 nm	0.699	0.543	0.433	0.562	0.492	0.394	0.295	0.422	0.516

Data Table S5. Colony formation SRB assay data used for calculations in paper.

Data Table S5. Colony formation SRB assay data used for calculations in paper.

	0 Gray					4 Gray			
	siNTC	siAMPKa1	siAkt1	siAMPKa1/Akt1		siNTC	siAMPKa1	siAkt1	siAMPKa1/Akt1
	0.843	0.635	0.457	0.556		0.143	0.193	0.151	0.124
	1.017	0.871	0.342	0.496		0.206	0.162	0.148	0.086
	0.848	0.771	0.5	0.395		0.19	0.106	0.113	0.116
	0.974	0.648	0.414	0.337		0.157	0.164	0.108	0.1
	0.729	0.665	0.431	0.353		0.233	0.192	0.14	0.161
	1.176	0.748	0.599	0.44		0.429	0.215	0.109	0.399
	0.822	0.619	0.38	0.571		0.172	0.228	0.118	0.143
	0.592	0.494	0.427	0.447		0.2	0.174	0.128	0.121
	0.67	0.75	0.386	0.422		0.18	0.165	0.113	0.122
	0.805	0.48	0.318	0.423		0.237	0.137	0.122	0.221
	0.929	0.887	0.338	0.563		0.246	0.182	0.092	0.331
	0.956	0.753	0.473	0.478		0.264	0.153	0.099	0.169
	0.825	0.964	0.432	0.761		0.196	0.139	0.134	0.15
	0.597	0.652	0.518	0.494		0.12	0.154	0.087	0.092
	0.714	0.872	0.304	0.443		0.132	0.227	0.083	0.104
	0.62	0.564	0.646	0.563		0.122	0.191	0.105	0.116
Absorbance at	0.744	0.772	0.338	0.4	Absorbance at	0.152	0.158	0.119	0.089
565 nm	0.679	0.737	0.358	0.768	565 nm	0.222	0.169	0.09	0.196
